# Metabolomics Reveals Flavonoid Profiles and Metabolite-Based Chemotypes in Plum (*Prunus salicina* Lindl.) Fruits with Different Colors

**DOI:** 10.3390/foods15173127

**Published:** 2026-09-03

**Authors:** Junwei Liu, Jiayi Li, Shengcheng Lin, Tingting Xie, Junning Gong, Jiaqi Zhang, Aoqi Xue, Shishi Luo, Yuanyuan Wang, Binqi Li, Sheng-Yen Wu, Faxing Chen, Honghong Deng

**Affiliations:** 1 College of Horticulture, Fujian Agriculture and Forestry University, Fuzhou 350002, China; 52403030062@fafu.edu.cn (J.L.); 3235201016@stu.fafu.edu.cn (J.L.); 3245201111@stu.fafu.edu.cn (S.L.); 12503005005@fafu.edu.cn (T.X.); 3245216069@stu.fafu.edu.cn (J.G.); 3245201025@stu.fafu.edu.cn (J.Z.); 12403005012@fafu.edu.cn (A.X.); 52403030030@fafu.edu.cn (S.L.); 52403030051@fafu.edu.cn (Y.W.); 62503030002@fafu.edu.cn (B.L.); fxchen@fafu.edu.cn (F.C.); 2 College of Plant Protection, Fujian Agriculture and Forestry University, Fuzhou 350002, China; sywu@fafu.edu.cn

**Keywords:** *Prunus salicina*, metabolomics, fruit color variation, flavonoid metabolic network, nutritional chemotype, UHPLC-MS/MS

## Abstract

Fruit color and phytochemical quality are essential commercial traits that determine the market value and consumer preference of plum (*Prunus salicina* Lindl.) fruits. Flavonoids are major bioactive phytochemicals closely associated with plum pigmentation and phytochemical properties; however, the metabolic mechanisms linking flavonoid variation to fruit color divergence remain unclear. Here, unbiased, widely targeted UHPLC MS/MS metabolomics was employed to comprehensively characterize tissue-specific (peel and flesh) and cultivar-dependent metabolic differences in three plum cultivars with distinct green, yellow, and red peel phenotypes. A total of 981 metabolites were successfully identified. Flavonoid biosynthesis was confirmed as the core pathway regulating tissue- and cultivar-specific metabolic divergence in plums. Reprogrammed flavonoid metabolic flux caused distinct accumulation of characteristic bioactive components, forming two distinct metabolite-based chemotypes: butein-dominant red plums and luteolin 7-glucoside-enriched yellow plums. Spatial metabolite profiling further confirmed the plum peel as the predominant tissue for flavonoid synthesis and accumulation, largely shaping phenotypic color differentiation and phytochemical variation. These findings clarify the intrinsic correlation between flavonoid profiles, visual appearance, and phytochemical characteristics of plum fruit, providing reliable metabolomic evidence for plum germplasm screening, quality evaluation, and resource utilization in the food industry.

## 1. Introduction

Plums (*Prunus* spp.), a cornerstone fruit crop in the Rosaceae family [1], are widely cultivated across temperate and subtropical regions of the Northern Hemisphere [2], where sufficient winter chilling is available to break dormancy. Domesticated at least 3000 years ago, they are among the earliest tree fruits managed by humans [3,4]. China is the world’s leading producer of plums, with an annual yield of approximately 6.9 million tons, accounting for about 54% of global production in 2024 [5].

Plums are valued not only for their low caloric content but also for their rich nutritional composition of sugars, proteins, dietary fibers, minerals, and vitamins. More importantly, they accumulate a variety of bioactive phytochemicals, including phenolics, flavonoids, anthocyanins, and carotenoids, which contribute to antioxidant, anti-inflammatory, anti-diabetic, anti-obesity, anti-apoptotic, and other health-promoting activities [6,7,8,9,10,11,12]. These attributes underpin their commercial importance in both fresh and processed markets (e.g., jam, marmalade, and brandy) and have stimulated interest in developing cultivars with enhanced functional properties. Beyond their agricultural and nutritional significance, from a food science perspective, the external appearance of fruit, particularly its color, serves as a primary visual cue that critically influences consumer preference, market competitiveness, and perceived fruit quality.

Globally, more than 6000 plum cultivars have been developed [13], derived from 19 to 40 species depending on taxonomic classification [14]. Among these, the diploid Chinese or Japanese plum (*P. salicina* Lindl.; 2n = 2x = 16) and the hexaploid European plum (*P. domestica* L.; 2n = 6x = 48) are the two most economically important species, encompassing a wide diversity of varieties [15]. Plums are considered one of the most genetically diverse deciduous fruit crops [16], and this diversity is particularly evident in the broad range of fruit colors, from green-yellow and red to purple, blue-violet, and black [17,18,19,20]. Fruit color is primarily attributed to flavonoids (especially anthocyanins), carotenoids, and chlorophylls [21]. Importantly, these pigments are not merely visual traits; they are direct reflections of specific phytochemical compositions that determine the nutritional and functional profile of the fruit as a food product. However, the comprehensive metabolic signatures associated with specific color classes remain poorly characterized.

While genomic, transcriptomic, and proteomic studies have identified key structural genes and regulatory networks involved in pigment biosynthesis [22], they cannot fully predict the final metabolic phenotype due to complex post-transcriptional, translational, and environmental influences [23]. In contrast, metabolomics enables the unbiased and quantitative profiling of small molecules, directly capturing the biochemical end result of these processes and providing the closest link to observable traits [22,24]. When combined with high-resolution LC-MS/MS platforms, widely targeted metabolomics allows for the simultaneous identification and quantification of hundreds to thousands of metabolites, making it a powerful tool for dissecting the metabolic basis of fruit color, flavor, and quality traits [22,24,25].

However, a systematic interpretation of color-related metabolic reprogramming is still lacking in plum research. In particular, it remains unclear whether distinct fruit color phenotypes correspond to stable metabolite-based subtypes that can support germplasm characterization. Therefore, this study employed UHPLC-MS/MS-based, unbiased, widely targeted metabolomics to analyze three plum cultivars representing green, yellow, and red color types. Our objectives were to: (1) decipher the metabolic network responsible for tissue-specific and cultivar-dependent color variation, and (2) identify and characterize key metabolite biomarkers associated with specific color phenotypes, thereby establishing a metabolite-based classification framework for plum phenotypic variation. This work provides systematic metabolomic insights into plum fruit quality variation and offers a fundamental reference for germplasm evaluation and resource utilization of colored plum cultivars.

## 2. Materials and Methods

### 2.1. Plant Materials and Sampling

**Figure 1:**
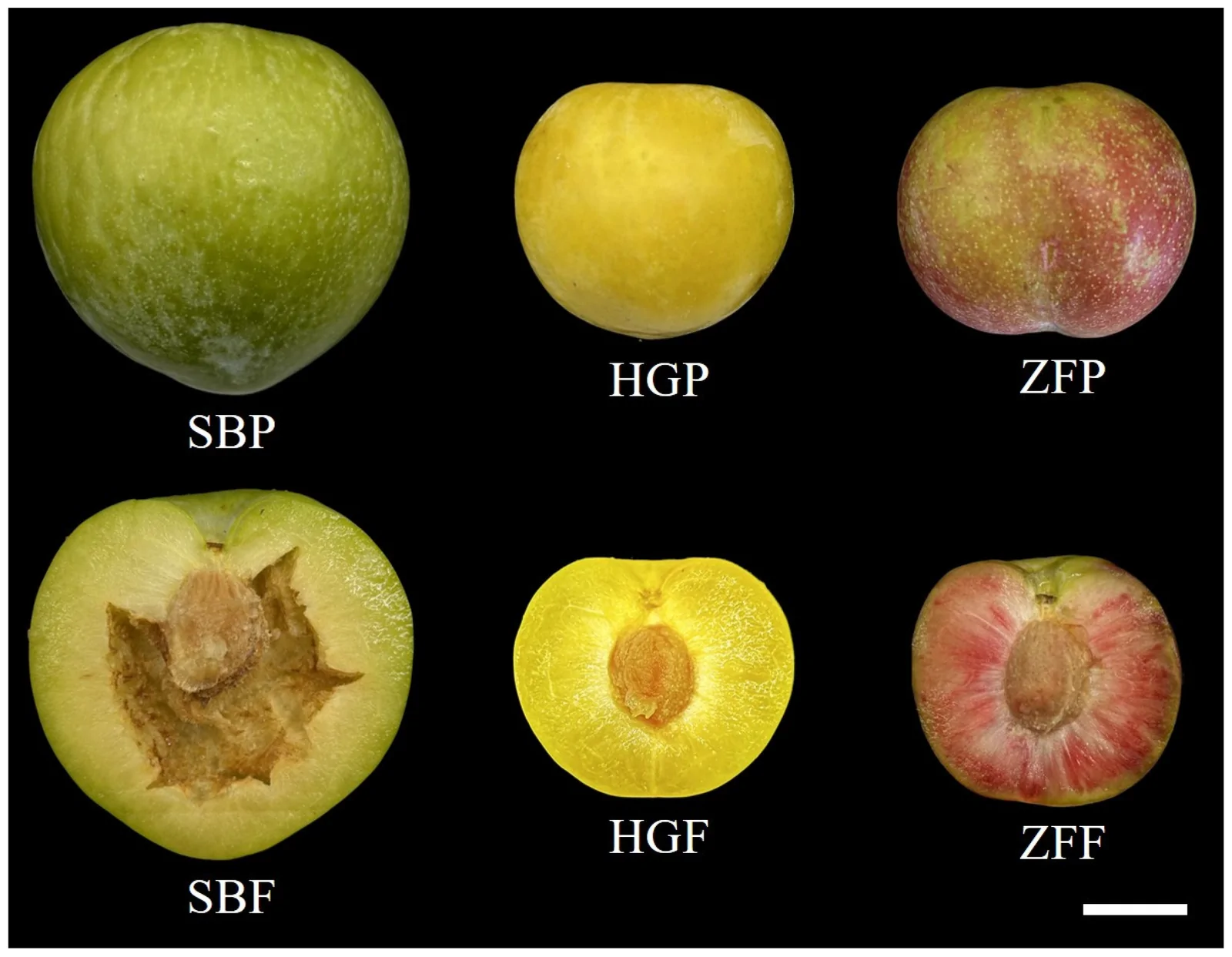
Fruit phenotypes of three Chinese plum (*Prunus salicina* Lindl.) cultivars with distinct peel and flesh colors. From left to right: ‘Shibanwannai’ (SB; green peel and flesh), ‘Huangguanli’ (HG; yellow peel and flesh), and ‘Zaofurong’ (ZF; red peel and flesh). Abbreviations: SBP, Shibanwannai peel; SBF, Shibanwannai flesh; HGP, Huangguanli peel; HGF, Huangguanli flesh; ZFP, Zaofurong peel; ZFF, Zaofurong flesh. Scale bar = 2 cm.

Three Chinese plum (*P. salicina* Lindl.) cultivars exhibiting distinct fruit color phenotypes were chosen for this study: ‘Shibanwannai’ (SB, green peel and flesh), ‘Huangguanli’ (HG, yellow peel and flesh), and ‘Zaofurong’ (ZF, red peel and flesh) (Figure 1). Trees were grown in a commercial block located in Shiban Village, Gutian County, Ningde City, Fujian, China (latitude: 26°39′3″ N, longitude: 118°49′4″ E). All scions were chip-budded onto *Prunus persica* L. Batsch rootstock, spaced at 3.5 m × 4 m, and maintained under uniform irrigation, fertilization and pest-control regimes.

Fruits were harvested at commercial maturity (firm-ripe, soluble solids 12–14°Brix) during the 2025 season. To eliminate canopy position effects, 36 sound fruits per cultivar (12 per tree × 3 trees) were picked randomly from upper, middle and lower thirds of the canopy between 08:00 and 10:00 h. Fruits were placed in insulated boxes with ice packs, transported to the laboratory within 1 h, and processed immediately.

Peel (≈1 mm thick) and flesh (inner mesocarp, excluding the stone) were separated with a ceramic blade pre-cooled in liquid nitrogen, snap-frozen in liquid nitrogen, ground to a fine powder with a mortar and pestle, and stored at −80 °C until analysis. For each cultivar—tissue combination, three independent pooled biological replicates were prepared. Each biological replicate consisted of pooled tissue powder derived from 12 individual fruits, which were randomly sampled across the three trees to reduce individual fruit variation.

### 2.2. Chemicals and Reagents

All solvents were HPLC or LC-MS grade. Methanol (MeOH, ≥99.0%) and the internal standard 2-amino-3-(2-chloro-phenyl)-propionic acid (98.0%) were purchased from Sigma-Aldrich (St. Louis, MO, USA). Ultrapure water (18.2 MΩ·cm at 25 °C) was generated with a Milli-Q water purification system (Millipore, Bedford, MA, USA) and filtered through a 0.22 μm membrane before use. All other chemicals were of analytical grade unless stated otherwise.

### 2.3. Metabolite Extraction for Widely Targeted Metabolomics

Frozen plum peel or flesh powder (pre-ground in liquid nitrogen with mortar and pestle) was kept at −80 °C until extraction. Aliquots (100 ± 2 mg) were weighed into 2 mL microtubes, immediately chilled in liquid nitrogen, and suspended in 600 µL ice-cold MeOH containing the internal standard 2-amino-3-(2-chloro-phenyl)-propionic acid (4 µg mL^−1^). Tubes were vortexed vigorously (30 s), sonicated in an ice-cooled ultrasonic bath (40 kHz, 25 °C, 15 min), and centrifuged at 12,000× *g* for 15 min at 4 °C. Supernatants were transferred to HPLC vials, passed through 0.22 µm PTFE syringe filters (Millipore, Bedford, MA, USA), and stored at −20 °C pending analysis.

A pooled quality-control (QC) sample was generated by combining 10 µL of every final extract; QC vials were inserted every 10 injections to monitor retention-time drift, signal intensity, and mass accuracy throughout the sequence.

### 2.4. UHPLC-MS/MS Conditions

Chromatographic separation was achieved on a Vanquish UHPLC system (Thermo Fisher Scientific, Waltham, MA, USA) equipped with an ACQUITY UHPLC HSS T3 column (2.1 × 100 mm, 1.8 μm; Waters, Milford, MA, USA) held at 40 °C. For positive ion acquisition, metabolites were eluted at 0.3 mL min^−1^ with a binary gradient of 0.1% formic acid in water (A) and 0.1% formic acid in acetonitrile (B), ramping from 10% B to 98% B between 1 and 5 min, holding at 98% B until 6.5 min, then returning to 10% B at 6.6 min and re-equilibrating for 1.4 min.

Negative-ion analysis used acetonitrile (C) versus 5 mM ammonium formate in water (D) under an identical gradient profile. Mass spectrometry was performed with an Orbitrap Exploris 120 mass spectrometer (Thermo Fisher Scientific) with a heated electrospray source. Fast polarity switching was applied to acquire both positive and negative ion modes within a single analytical run. The instrument was set to full MS–data-dependent MS^2^ mode: spray voltages +3.5 kV/−2.5 kV, capillary temperature 325 °C, sheath gas 40 arbitrary units, auxiliary gas 10 arbitrary units, full scans m/z 100–1000 at 60,000 FWHM, and up to four MS^2^ events per cycle at 15,000 FWHM with 30% normalized collision energy and 8 s dynamic exclusion to maximize metabolite coverage while minimizing redundant fragmentation [26].

### 2.5. Data Preprocessing

Raw UHPLC-MS/MS data were first converted to mzXML format with the MSConvert module of ProteoWizard (v3.0.8789) [27] and subsequently processed by XCMS package (v3.12.0) running under R software (v4.1.0) [28]. Feature detection, retention-time alignment, and peak grouping were performed with the centWave algorithm using a mass tolerance of 15 ppm, a peak width range of 5–30 s, and a minimum m/z increment (mzdiff) of 0.01. Matrix-wide batch effects were removed by QC-based robust LOESS correction; only features exhibiting a relative standard deviation (RSD) < 30% across QC injections were retained for statistical analysis [26].

### 2.6. Metabolite Annotation

Metabolites were identified by combining accurate-mass data and MS/MS spectra acquired in positive- and negative-ion modes. Parent-ion m/z values were first extracted and deconvoluted; elemental compositions were predicted within a 5 ppm window while accounting for common adducts ([M + H]^+^, [M + Na]^+^, [M − H]^−^, etc.). These formulas were matched against the HMDB, MassBank, KNApSAcK, ReSpect, LipidMaps, and KEGG databases to obtain putative MS-level identifications. For each candidate, experimental MS/MS fragmentation patterns (HCD, 30% NCE) were compared with reference spectra in the public databases. Annotation required at least three diagnostic fragment ions and a spectral mirror-match score ≥ 0.7. Only compounds that satisfied both accurate-mass tolerance and spectral similarity thresholds were retained as putatively annotated metabolites corresponding to Metabolomics Standards Initiative (MSI) Level 2 identification.

### 2.7. Statistical Data Analysis

Multivariate pattern recognition was performed with the Ropls package (v1.22.0) in R software [29]. After Pareto scaling, unsupervised principal component analysis (PCA) was first run to detect intrinsic clustering and outlier behavior. Supervised partial least-squares discriminant analysis (PLS-DA) was used as the primary model for multi-group discrimination among plum groups. Orthogonal projection to latent structures discriminant analysis (OPLS-DA) was further applied as a complementary approach to separate group-related metabolic variation from orthogonal noise. Both models were calculated to maximize class separation between color groups. Model validity was verified by 7-fold cross-validation and 200-times permutation testing (Q^2^ > 0.5, *p* < 0.01). Differential metabolites were screened by combining multivariate and univariate statistics. For multi-group datasets, one-way ANOVA was used for overall group comparison, followed by Student’s *t*-test for pairwise comparisons with FDR correction for multiple testing. Metabolites satisfying VIP > 1 from multivariate models and *p* < 0.05 from univariate tests were regarded as differentially abundant metabolites. Considering the limited three biological replicates per group, all multivariate classification outputs are interpreted as exploratory metabolic evidence rather than definitive proof of group separation.

### 2.8. Pathway Analysis

MetaboAnalyst 6.0 [30] (https://www.metaboanalyst.ca) was used to integrate pathway enrichment and topology analysis. Briefly, differential metabolites (KEGG IDs) were mapped against reference pathways (e.g., ko00941, ko00943, ko00944, ko00946, et al.). Over-representation was tested with a hypergeometric test, and pathway impact was calculated from relative-betweenness centrality. Pathways with FDR < 0.05 and impact > 0.1 were deemed biologically significant and visualized with KEGG Mapper.

## 3. Results

### 3.1. Quality Control and Data Reliability of the Metabolomics Dataset

**Figure 2:**
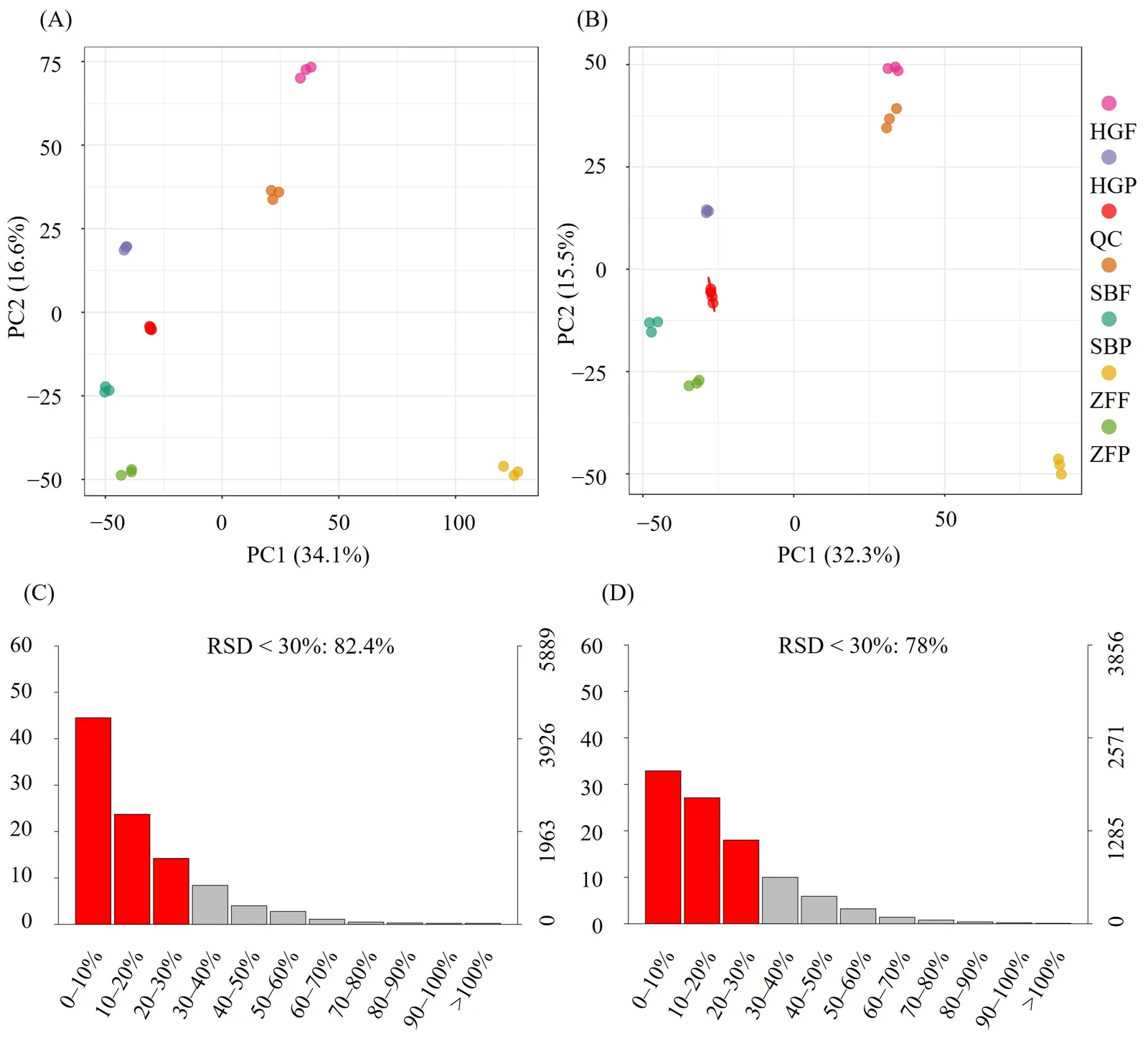
Evaluation of data quality in metabolomics. PCA score plot of QC samples from positive (**A**) and negative (**B**) ionization modes, showing tight clustering. Histograms displaying the RSD distribution of metabolic features in positive (**C**) and negative (**D**) ionization modes after QA filtering, with the majority of features exhibiting an RSD below 30%.

Quality control analyses confirmed the high reliability of the metabolomics data. The overlaid base peak chromatograms showed excellent overlap among QC samples (Figure S1). PCA score plots demonstrated tight clustering of QC samples in both positive (Figure 2A) and negative (Figure 2B) ionization modes, indicating excellent instrumental stability and methodological reproducibility throughout the entire analytical sequence. After quality assurance filtering, 82.4% and 78.0% of metabolic features exhibited a relative standard deviation (RSD) below 30% in positive (Figure 2C) and negative (Figure 2D) ionization modes, respectively. These results collectively confirm the high data quality required for subsequent differential metabolite analyses.

### 3.2. Robustness of Multivariate Statistical Models

To evaluate the performance of subsequent differential metabolite screening, both PLS-DA (Table 1) and OPLS-DA (Table 2) models were constructed and subjected to standard validation procedures. High cumulative Y-matrix explanation (R2Y(cum) close to 1.000) and high predictive parameter Q2(cum) (>0.946) were observed across most comparisons for both ionization modes (Table 1 and Table 2). PLS-DA and OPLS-DA are employed here primarily to visualize metabolic separation trends, while the identification of DAMs relies on univariate statistical testing.

### 3.3. Global Metabolite Annotation and Composition

**Figure 3:**
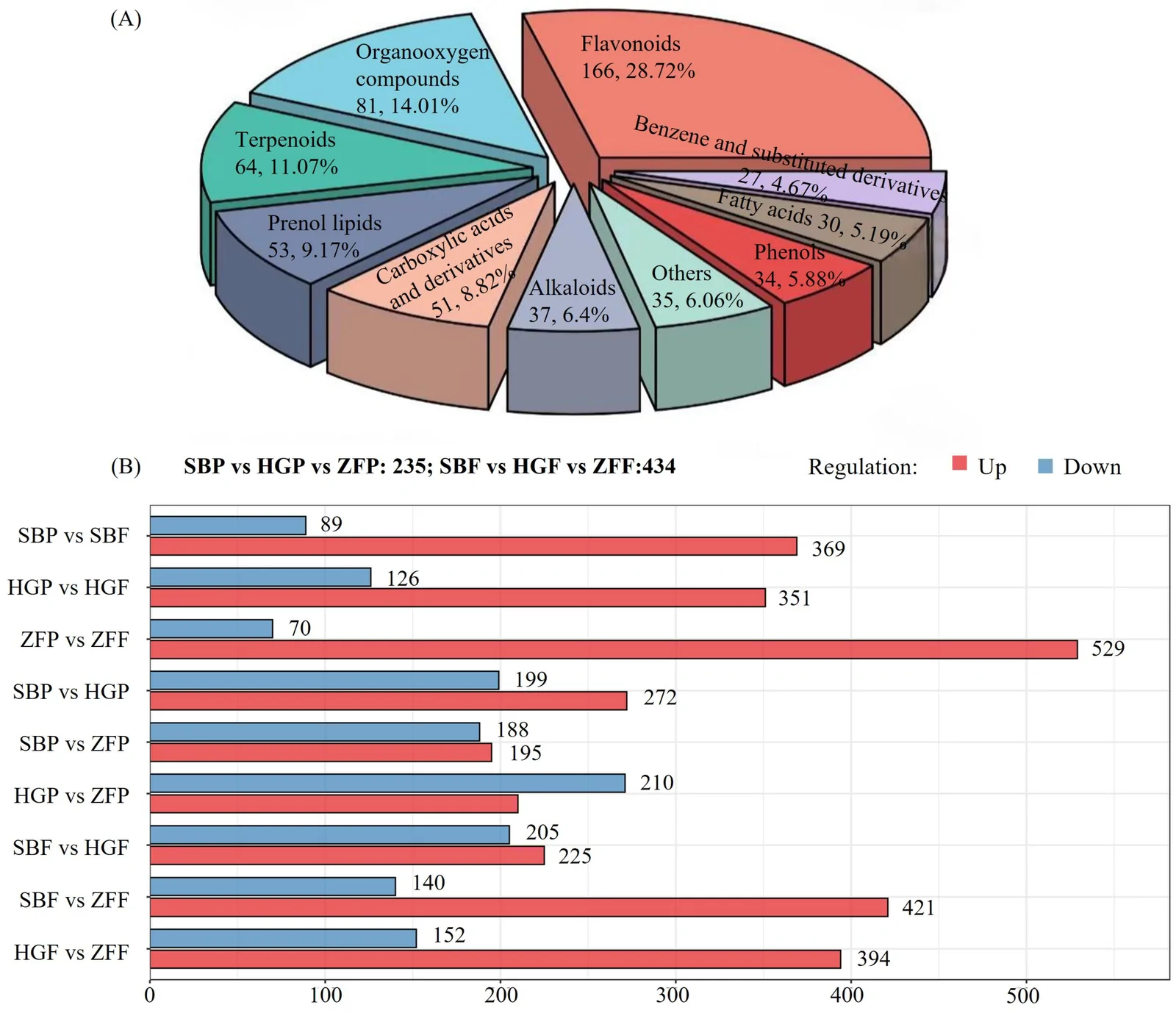
Overview of annotated and differentially abundant metabolites. (**A**) Pie chart showing the distribution of the 981 annotated metabolites across major chemical classes. (**B**) Bar plot summarizing the number of differentially abundant metabolites (DAMs) identified in all pairwise comparisons.

Metabolite annotation was achieved by matching the accurate mass, retention time, and MS/MS spectra of the detected features against a plant-specific metabolome database. This workflow successfully annotated 981 metabolites in the plum samples, and the relative abundance of these annotated metabolites across all samples is summarized in Table S1. The dataset encompassed a diverse range of compound classes, with flavonoids constituting the most predominant group (166 metabolites, 28.72%), followed by organooxygen compounds (81, 14.01%), terpenoids (64, 11.07%), prenol lipids (53, 9.17%), carboxylic acids and derivatives (51, 8.82%), alkaloids (37, 6.4%), phenols (34, 5.88%), fatty acyls (30, 5.19%), benzene and substituted derivatives (27, 4.67%), and other compounds (35, 6.06%) (Figure 3A).

### 3.4. Overview of Differentially Abundant Metabolites

To identify differentially abundant metabolites (DAMs), metabolites with confident Level 2 annotations were selected and subjected to both univariate (*p*-value) and multivariate analyses. DAMs were defied as metabolites satisfying the thresholds of *p*-value < 0.05 and VIP ≥ 1.0 were defined as DAMs. A comprehensive set of pairwise comparisons was performed, including tissue-specific comparisons (peel vs. pulp within each cultivar) and cross-cultivar analyses (for peel and flesh tissues separately) (Figure 3B).

The number of DAMs varied considerably across different comparisons. For instance, the “ZFP vs. ZFF” comparison yielded the highest number of total DAMs (599), whereas the “SBP vs. ZFP” comparison resulted in the fewest (383). Notably, the three-cultivar comparison of flesh tissues (SBF vs. HGF vs. ZFF) identified 434 common DAMs, suggesting substantial and systemic metabolic divergence among cultivars in the edible portion of the fruit. Detailed identification results for the DAMs in each comparison group are provided in Table S2.

### 3.5. Key DAMs Underlying Tissue and Cultivar Divergence

**Figure 4:**
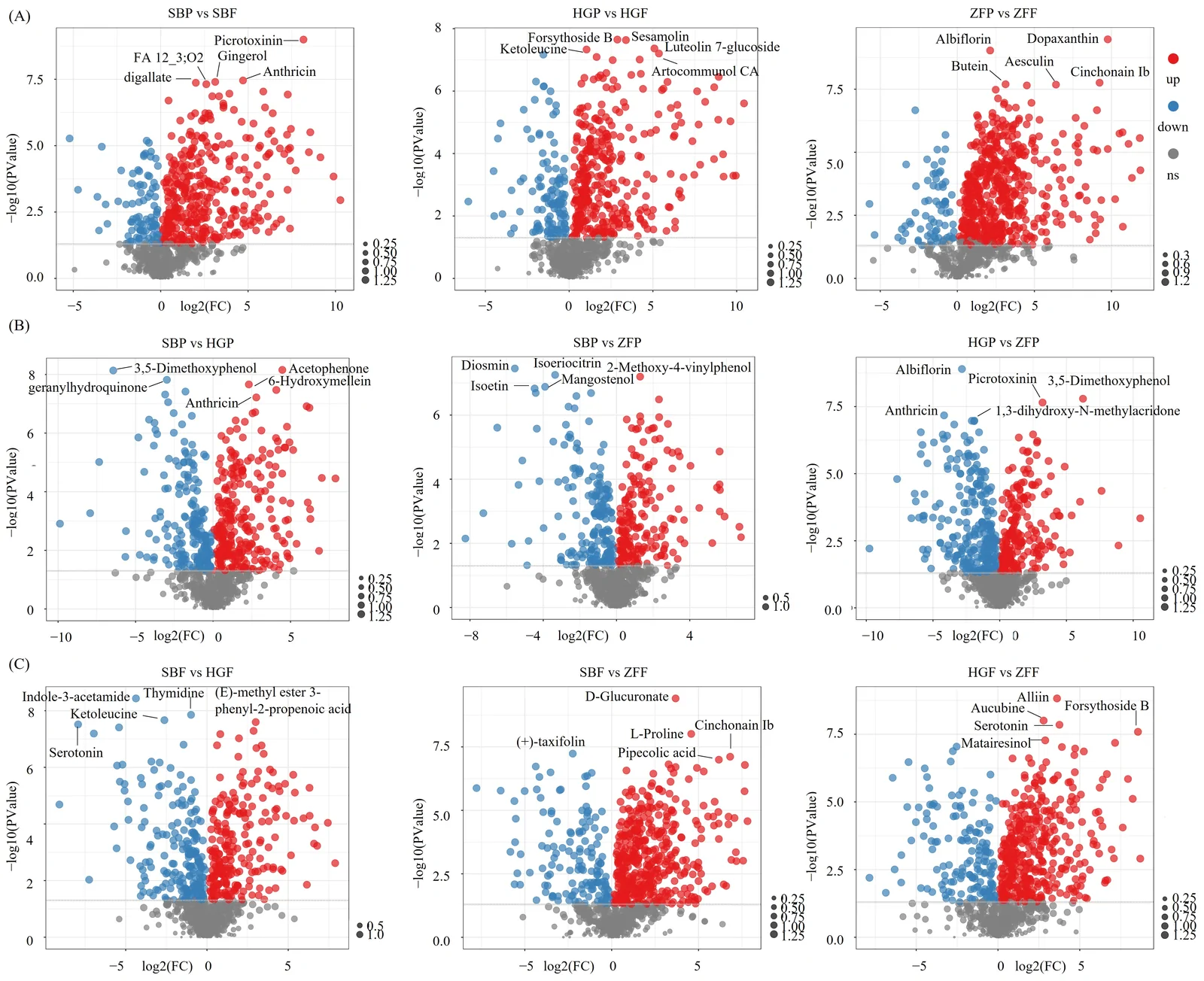
Volcano plots of differentially abundant metabolites (DAMs) in all pairwise comparisons. (**A**) Tissue-specific comparisons (peel vs. flesh within each cultivar). (**B**) Cross-cultivar comparisons of peel tissues. (**C**) Cross-cultivar comparisons of flesh tissues. In all plots, the *x*-axis represents the log2-transformed fold change (FC), and the *y*-axis represents the −log10-transformed *p*-value. Each point corresponds to a metabolite, with its size proportional to the Variable Importance in Projection (VIP) score. Metabolites meeting the significance thresholds (FC ≥ 2 or FC ≤ 0.5, *p*-value < 0.05, and VIP ≥ 1) are colored red (up-regulated in the first-named group) or blue (down-regulated in the first-named group). Grey points represent non-significant metabolites.

Metabolic profiling revealed substantial divergence between peel and flesh tissues across three cultivars. In the green-fruited ‘Shibanwannai’ (SBP vs. SBF), specialized metabolites such as picrotoxinin and anthricin were markedly accumulated in the peel (FC = 288.61 and 26.23, respectively). Similarly, the peel of the yellow-fruited ‘Huangguanli’ (HGP vs. HGF) showed pronounced enrichment of phenylpropanoids, including forsythoside B (FC = 7.36) and sesamolin (FC = 10.51), along with the flavonoid luteolin 7-glucoside (FC = 34.03). The most striking tissue-specific pattern was observed in the red-fruited ‘Zaofurong’ (ZFP vs. ZFF), where peel tissues exhibited extreme accumulation of pigments and antioxidants, including dopaxanthin (FC = 867.52), cinchonain Ib (FC = 601.32), and butein (FC = 8.75). These findings underscore the peel’s role as the primary site for the synthesis of protective and pigmented compounds (Figure 4A).

Cross-cultivar comparison of peel tissues further identified metabolites strongly associated with specific fruit colors. Green peels were characterized by elevated levels of acetophenone (FC = 22.21 in SBP) and 6-hydroxymellein, whereas yellow peels accumulated higher levels of geranylhydroquinone. In contrast, the red peel of ‘Zaofurong’ was distinctly enriched with flavonoids such as diosmin, isoeriocitrin, and isoetin. Additionally, metabolites including albiflorin and picrotoxinin were more abundant in red compared to yellow peels, while 3,5-dimethoxyphenol was uniquely elevated in yellow peels (Figure 4B).

Analysis of flesh tissues also revealed cultivar-specific metabolic signatures. The flesh of the yellow cultivar was distinguished by high levels of indole-3-acetamide and serotonin, whereas the green flesh showed greater abundance of (E)-methyl ester 3-phenyl-2-propenoic acid. Red flesh generally exhibited lower abundance of specialized metabolites, though key compounds such as L-proline and cinchonain Ib were more abundant in green and yellow flesh. Notably, serotonin levels were significantly higher in yellow than in red flesh (Figure 4C).

### 3.6. Pathway Analysis Highlights Flavonoid Biosynthesis as a Conserved Regulatory Axis

**Figure 5:**
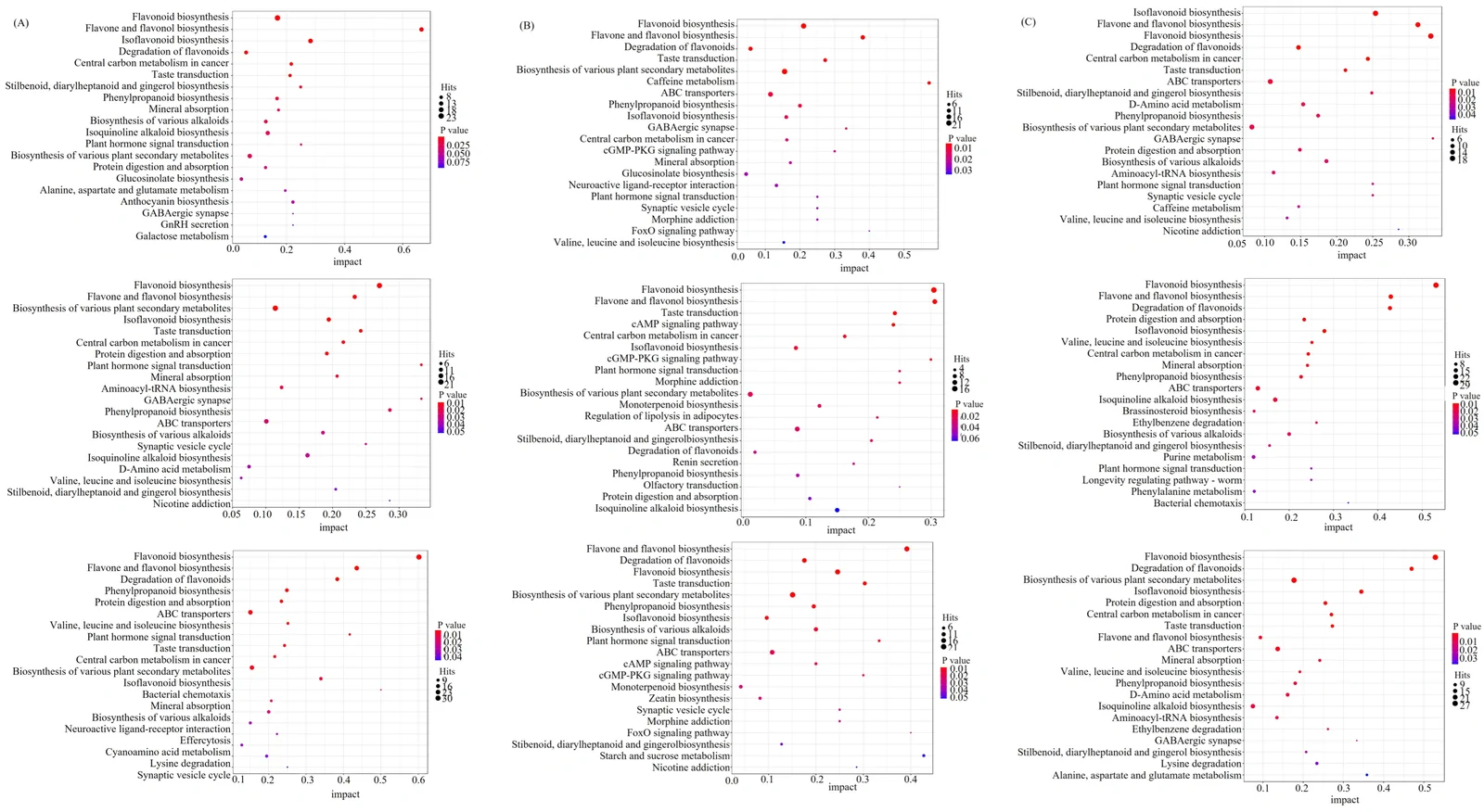
KEGG pathway enrichment analysis of differentially abundant metabolites. Subpanel (**A**) shows enrichment results for peel versus flesh comparisons within each cultivar; (**B**) shows cross-cultivar comparisons of peel tissues; and (**C**) shows cross-cultivar comparisons of flesh tissues. Each dot within a subpanel represents an enriched pathway obtained from one individual comparison. The y-axis represents the enriched KEGG pathways, and the x-axis represents the IMPACT value from pathway topology analysis. The size of the dots corresponds to the number of differentially abundant metabolites mapped to the respective pathway. The color of the dots corresponds to the −log10-transformed *p*-value, where a redder hue indicates a smaller *p*-value and a bluer hue indicates a larger *p*-value.

KEGG pathway enrichment analysis revealed that the flavonoid biosynthesis pathway (ko00941) was the most significantly and consistently enriched pathway across all biological comparisons. This included tissue-specific (peel vs. flesh) comparisons (Figure 5A), all cross-cultivar pairwise comparisons, and the consolidated three-way cultivar analyses of both peel (Figure 5B) and flesh (Figure 5C) tissues.

The “ZFP vs. ZFF” comparison showed the most profound enrichment for flavonoid biosynthesis (*p* = 2.70 × 10^−16^, 30 DAMs), followed by “SBP vs. SBF” (*p* = 1.29 × 10^−12^, 23 DAMs) and “HGP vs. HGF” (*p* = 1.50 × 10^−9^, 20 DAMs) (Figure 5A). In all three cultivars, the vast majority of these flavonoid DAMs were up-regulated in the peel compared to the flesh (Figure 5A), strongly suggesting that flavonoids are predominantly synthesized and accumulated in the outer tissue of the fruit.

This consistent KEGG enrichment pattern persisted in cross-cultivar comparisons, further linking varietal color differences to divergent flavonoid metabolism. For instance, the “SBF vs. ZFF” (*p* = 7.95 × 10^−16^, 29 DAMs) and “HGF vs. ZFF” (*p* = 3.16 × 10^−14^, 27 DAMs) comparisons in the flesh tissues, as well as the “SBP vs. HGP” (*p* = 1.24 × 10^−9^, 20 DAMs) comparison in the peel tissues, all highlighted flavonoid biosynthesis as the top enriched pathway (Figure 5B,C).

### 3.7. Network Analysis Reveals Potential Metabolic Flux Divergence

**Figure 6:**
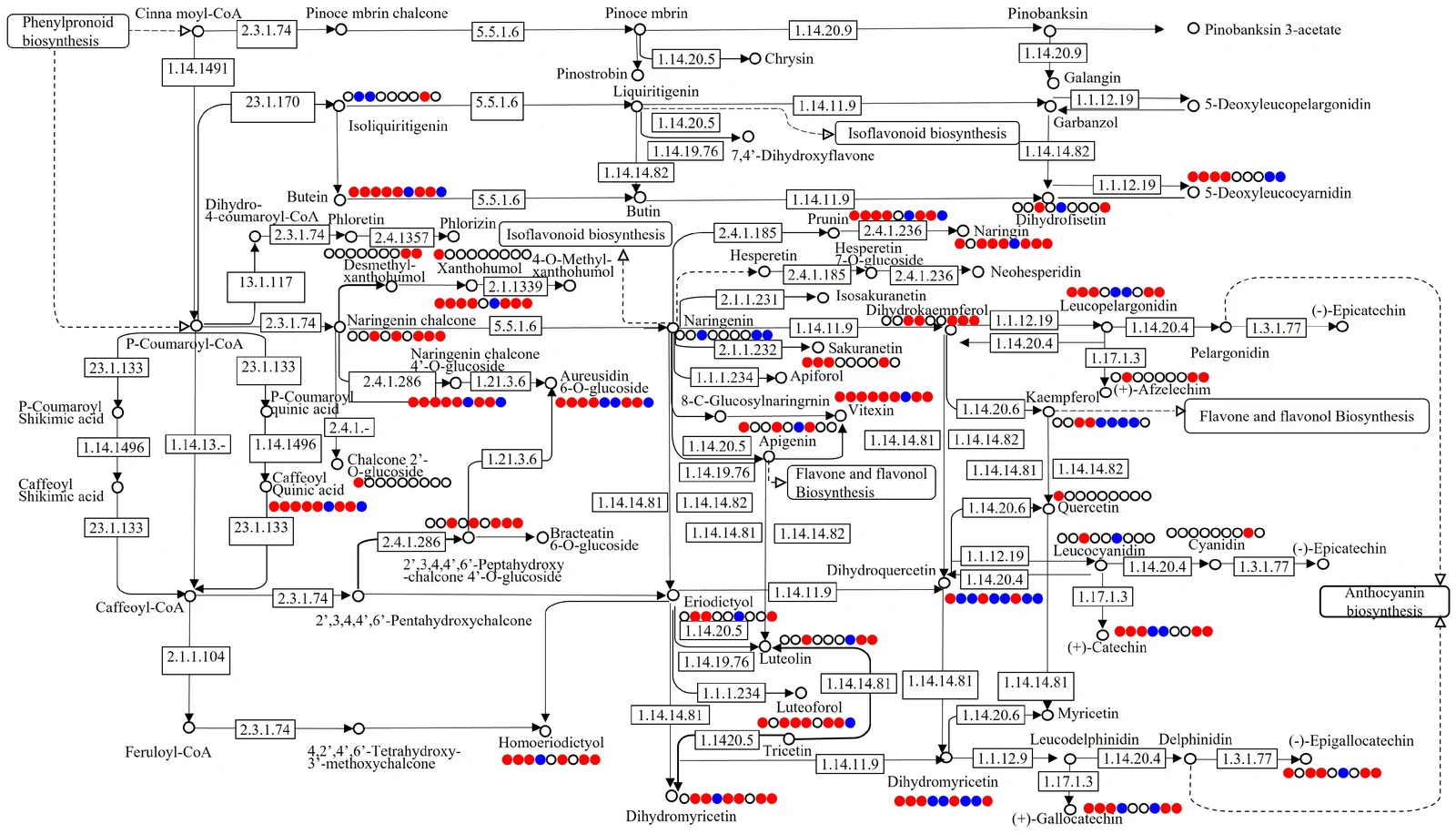
Visualization of the flavonoid biosynthesis pathway (ko00941) mapped with differentially abundant metabolites (DAMs). Rectangles represent protein/enzyme molecules, and circles represent metabolite molecules. Metabolites are highlighted in red (significantly up-regulated) or blue (significantly down-regulated) based on the differential abundance analysis. From left to right, the mapped comparisons are: peel vs. flesh within cultivars, cross-cultivar comparison of peel tissues, and cross-cultivar comparison of flesh tissues, specifically: SBP vs. SBF, HGP vs. HGF, ZFP vs. ZFF, SBP vs. HGP, SBP vs. ZFP, HGP vs. ZFP, SBF vs. HGF, SBF vs. ZFF, and HGF vs. ZFF.

Comprehensive mapping of DAMs onto the flavonoid biosynthesis pathway provided an integrated visualization of coordinated metabolic changes and their regulatory roles (Figure 6). This map reveals consistent and contrasting accumulation patterns of structurally related metabolites between cultivars. Specifically, the peel of the red-fruited ‘Zaofurong’ showed pronounced accumulation of anthocyanin-related compounds, whereas the peel of the yellow-fruited ‘Huangguanli’ was characterized by dominant accumulation of flavones and flavonols. These distinct profiles, which involve both upstream precursors and downstream derivatives, demonstrate a systematic reprogramming of metabolite levels across the entire pathway network.

Beyond the core flavonoid biosynthesis pathway, the enrichment analysis consistently identified several interconnected pathways within the broader phenylpropanoid metabolic grid (Figure 5). These included flavone and flavonol biosynthesis (ko00944, Figure S2), isoflavonoid biosynthesis (ko00943, Figure S3), and notably, flavonoid degradation (ko00946, Figure S4). The recurrent co-enrichment of these specific sub-pathways across all tissue and cultivar comparisons indicates that the observed metabolic divergence is not an isolated event but is embedded within a wider, coordinated metabolic system.

## 4. Discussion

### 4.1. The Flavonoid Metabolic Network as a Central Determinant of Plum Fruit Phenotype

In this study, we performed unbiased, widely targeted metabolomic profiling across three plum cultivars with distinct fruit colors to systematically characterize tissue- and genotype-dependent metabolic variation. Rather than targeting predefined pigment compounds, this untargeted-like global metabolite screening enabled an objective and comprehensive comparison of metabolic differences underlying divergent color phenotypes. This systems-level metabolomic analysis advances our understanding of plum fruit color formation beyond individual compositional changes, filling the knowledge gap regarding the core metabolic basis of color differentiation and its association with fruit phytochemical characteristics.

This integrated metabolomics study advances our understanding of plum fruit color formation beyond individual compositional changes, filling the knowledge gap regarding the core metabolic basis of color variation and its link to phytochemical quality. Collectively, the results demonstrate that the phenylpropanoid pathway, dominated by flavonoid biosynthesis, functions as the central regulatory axis determining both tissue specialization and cultivar-dependent pigmentation in plum fruits.

The foundational role of this network is first evidenced by flavonoids constituting the most abundant metabolite class in our dataset (166 metabolites,.28.27%), corroborating plum’s well-established status as a rich source of these bioactive compounds [31]. Critically, the consistent and significant enrichment of the flavonoid biosynthesis pathway (ko00941) across every biological comparison, including tissue-specific (peel vs. flesh) and all cross-cultivar analyses (Figure 5), unequivocally establishes it as a fundamental metabolic grid. This finding resonates with and functionally extends genomic insights into the essential roles of these pathways during plum domestication [32], which also aligns with prior metabolomic studies on *Prunus* species that highlight flavonoids as core phytochemical components contributing to fruit color, flavor, and bioactivity [33].

### 4.2. A Metabolic Flux Reprogramming Hypothesis for Cultivar-Specific Coloration

A key insight from our data is the systematic reprogramming of the flavonoid network output, which directly underlies cultivar-specific color phenotypes. The visualization of coordinated metabolite changes (Figure 6) reveals contrasting accumulation patterns between the three plum cultivars, linking specific metabolites to distinct color traits: the red peel of ‘Zaofurong’ is characterized by extreme accumulation of pigments such as butein, while the yellow peel of ‘Huangguanli’ is dominated by luteolin 7-glucoside. These distinct, cultivar-specific profiles, encompassing shifts in both shared upstream precursors and downstream derivatives, lead us to propose a testable hypothesis: the observed color divergence is driven by differential routing of metabolic flux at key branch points within the flavonoid network.

This model of “branch-point control” provides a mechanistic framework that aligns with and refines the existing model where peel color is determined by specific pigment composition [18,34], moving beyond mere cataloguing of metabolic differences to explaining their potential origin. This flux reprogramming is further supported by the consistent co-enrichment of interconnected pathways (flavone and flavonol biosynthesis, isoflavonoid biosynthesis, and flavonoid degradation) within the broader phenylpropanoid metabolic grid, indicating that color variation is part of a coordinated systemic metabolic response.

### 4.3. Synergistic Pigmentation and the Functional Role of Key Metabolites

Our analysis suggests that the final color phenotype of plum fruit may be a synergistic outcome of multiple metabolites, rather than a single pigment. Notably, compounds such as butein and luteolin 7-glucoside are recognized not only as pigments and co-pigments but also as biomarkers for distinct metabolic profiles [10]. This positions them as more than compositional markers; they are indicators of underlying biochemical differences that may have broader physiological and nutritional significance.

Specifically, butein, a flavonoid reported to exhibit potential anti-diabetic, anti-cancer, and anti-inflammatory, and antioxidant effects [35], is uniquely enriched in the red cultivar, while luteolin 7-glucoside, a flavone with putative antioxidant, neuroprotective, and vascular protective activities in external research [36], dominates the yellow cultivar. Additionally, our study identified several novel metabolite biomarkers associated with specific color phenotypes, such as acetophenone and 6-hydroxymellein in green peels, and geranylhydroquinone in yellow peels, compounds not previously linked to plum color variation, which may serve as novel markers for cultivar identification and breeding.

### 4.4. The Peel as a Dynamic and Specialized Metabolic Compartment

The universal upregulation of flavonoids in the peel across all three cultivars (Figure 4A) reinforces its role as a primary specialized biosynthetic and protective compartment [37], a pattern consistently observed for antioxidants and phenolics in plums [10,12,38,39,40]. Our results further highlight the peel’s functional significance: in the red-fruited ‘Zaofurong’, peel tissues exhibited extreme accumulation of pigments and antioxidants including dopaxanthin, cinchonain Ib, and butein; in the yellow ‘Huangguanli’, peel was enriched in phenylpropanoids and luteolin 7-glucoside; and in the green ‘Shibanwannai’, peel accumulated specialized metabolites such as picrotoxinin and anthricin. Importantly, the recurrent enrichment of the flavonoid degradation pathway (ko00946) alongside biosynthesis suggests the peel is a site of dynamic metabolic turnover [41], indicating that color may be actively fine-tuned and linked to broader physiological and environmental responses. The health-promoting potential of these plum cultivars is underscored by the significant enrichment of these bioactive metabolites, whose antioxidant activities are well-documented [6,8,9,11,12,42,43].

### 4.5. Implications for Metabolite-Based Chemotypes and Food Nutritional Characterization

Our metabolomic profiling establishes that distinct plum color phenotypes correspond to specific metabolite-based chemotypes, marked by dominant health-associated metabolites: a butein-rich red chemotype and a luteolin 7-glucoside-rich yellow chemotype. These signature compounds are not merely pigments; they are well-documented bioactive agents with reported health-promoting properties according to published literature [35,36], which are further supported by the tissue-specific accumulation patterns observed in the study. This chemotype framework offers a metabolite-based reference for plum germplasm evaluation and food ingredient development.

## 5. Conclusions

In conclusion, this study systematically characterized tissue-specific and cultivar-dependent metabolic profiles of differently colored plum fruits via unbiased, widely targeted metabolomics. The results clarified that flavonoid biosynthesis serves as the core metabolic pathway responsible for fruit color differentiation among plum cultivars. Two distinct metabolite-based chemotypes were successfully defined: red plums characterized by dominant butein accumulation and yellow plums enriched in luteolin 7-glucoside. Spatial metabolite profiling further verified that plum peel functions as the major tissue for flavonoid biosynthesis and accumulation, primarily shaping the metabolic and phenotypic characteristics of plum fruits. The present results elucidate the inherent relationship between flavonoid metabolic signatures and fruit color phenotypes, providing solid metabolomic evidence for plum germplasm characterization and quality evaluation.

## Figures and Tables

**Table 1:** Validation parameters of PLS-DA model.

Comparison	Pre	Positive Ion Mode			Negative Ion Mode		
		R2X (cum)	R2Y (cum)	Q2 (cum)	R2X (cum)	R2Y (cum)	Q2 (cum)
SBP vs. SBF	2	0.7	1	0.988	0.729	1	0.991
HGP vs. HGF	2	0.71	1	0.989	0.696	1	0.987
ZFP vs. ZFF	2	0.811	1	0.997	0.766	1	0.997
SBP vs. HGP	2	0.687	1	0.982	0.69	1	0.987
SBP vs. ZFP	2	0.678	1	0.983	0.649	1	0.977
HGP vs. ZFP	2	0.688	1	0.988	0.684	1	0.983
SBF vs. HGF	2	0.655	1	0.983	0.636	1	0.97
SBF vs. ZFF	2	0.738	1	0.991	0.717	1	0.987
HGF vs. ZFF	2	0.739	1	0.99	0.715	1	0.988
SBP vs. HGP vs. ZFP	2	0.664	1	0.973	0.653	1	0.981
SBF vs. HGF vs. ZFF	2	0.725	0.999	0.996	0.685	1	0.997

Note: pre, number of principal components; R2X, model interpretability for the X-variable matrix; R2Y, model interpretability for the Y-variable matrix; Q2, model predictability.

**Table 2:** Validation parameters of OPLS-DA model.

Comparison	Pre	Positive Ion Mode			Negative Ion Mode		
		R2X (cum)	R2Y (cum)	Q2 (cum)	R2X (cum)	R2Y (cum)	Q2 (cum)
SBP vs. SBF	1 + 1 + o	0.7	1	0.977	0.729	1	0.98
HGP vs. HGF	1 + 1 + o	0.71	1	0.979	0.696	1	0.977
ZFP vs. ZFF	1 + 1 + o	0.811	1	0.993	0.766	1	0.989
SBP vs. HGP	1 + 1 + o	0.687	1	0.978	0.69	1	0.975
SBP vs. ZFP	1 + 1 + o	0.678	1	0.963	0.649	1	0.957
HGP vs. ZFP	1 + 1 + o	0.688	1	0.978	0.684	1	0.967
SBF vs. HGF	1 + 1 + o	0.655	1	0.959	0.636	1	0.962
SBF vs. ZFF	1 + 1 + o	0.738	1	0.985	0.717	1	0.975
HGF vs. ZFF	1 + 1 + o	0.739	1	0.983	0.715	1	0.983
SBP vs. HGP vs. ZFP	1 + 1 + o	0.664	1	0.946	0.653	1	0.926
SBF vs. HGF vs. ZFF	1 + 1 + o	0.725	0.999	0.974	0.685	1	0.964

Note: pre, number of principal components; R2X, model interpretability for the X-variable matrix; R2Y, model interpretability for the Y-variable matrix; Q2, model predictability.

## Data Availability

The original contributions presented in this study are included in the article/Supplementary Materials. Further inquiries can be directed to the corresponding author.

## Supplementary Materials

The following supporting information can be downloaded at: https://www.mdpi.com/article/10.3390/foods15173127/s1, Figure S1: Representative base peak chromatograms (BPC) acquired in (A) positive and (B) negative ionization modes; Figure S2: KEGG pathway enrichment analysis of flavone and flavonol biosynthesis (ko00944); Figure S3: KEGG pathway enrichment analysis of isoflavonoid biosynthesis (ko00943); Figure S4: KEGG pathway enrichment analysis of flavonoid degradation (ko00946); Table S1: List of metabolite identification and quantification; Table S2: List of differentially abundant metabolites.
